# Update on R-loops in genomic integrity: Formation, functions, and implications for human diseases

**DOI:** 10.1016/j.gendis.2024.101401

**Published:** 2024-08-30

**Authors:** Min Zhu, Xinyu Wang, Hongchang Zhao, Zhenjie Wang

**Affiliations:** a Department of Emergency Surgery, The First Affiliated Hospital of Bengbu Medical University, Bengbu, Anhui 233004, China; b Institute of Emergency and Critical Care, The First Affiliated Hospital of Bengbu Medical University, Bengbu, Anhui 233004, China; c Institute for Translation Medicine, The Affiliated Hospital of Qingdao University, College of Medicine, Qingdao University, Qingdao, Shandong 266021, China

**Keywords:** DNA repair, Double strand breaks, Genome instability, Immune response, R-loops, Replication stress

## Abstract

R-loops, three-strand nucleic acid structures, have emerged as crucial players in various physiological processes, including the regulation of gene expression, DNA replication, and class switch recombination. However, their presence also poses a significant threat to genome stability. A particularly challenging aspect is understanding the dynamic balance between R-loops' “light” and “dark” sites, especially concerning maintaining genome integrity. The complex and multifaceted roles of R-loops in genome stability necessitate a deeper understanding. This review offers a comprehensive exploration of the formation, resolution, and implications of R-loops, particularly in the context of DNA damage and human disease. We delve into the dualistic nature of R-loops, highlighting their role in DNA damage response and repair, and discuss the therapeutic potential arising from our evolving understanding of these enigmatic entities. Emphasizing recent advancements and unresolved questions, this review aims to provide a cohesive overview of R-loops, inviting further inquiry and investigation into their complex biological significance.

## Introduction

R-loops are non-B form DNA structures predominantly formed during transcription, crucial for gene transcription and DNA replication.^1^, ^2^, ^3^, ^4^, ^5^ Emerging evidence suggests that abnormal R-loop levels interfere with DNA replication, repair, and transcription, leading to replication stress, DNA breakage, and genomic instability.^3^^,^^4^^,^^6^^,^^7^ Transcriptional complexes serve as endogenous barriers that often impede replication forks due to competition for the same DNA template. Abnormal R-loop accumulation during transcription can impede ongoing replication, resulting in transcription-replication collisions. Notably, head-on transcription-replication collisions — and less so co-directional collisions — lead to frequent replication stalling and damage.^8^, ^9^, ^10^, ^11^, ^12^, ^13^ Therefore, R-loop-induced transcription-replication collisions are widely accepted as a major source of genome instability *in vivo*.^14^ Given these complex biological processes, understanding the mechanisms of R-loop formation and their specific functions is crucial for gaining deeper insights into their role in genome stability.

Double-strand breaks (DSBs) represent the most severe form of DNA damage as they affect both DNA strands. DSBs can be induced by chemical agents, ionizing radiation, or endogenous toxic agents.^15^, ^16^, ^17^, ^18^, ^19^, ^20^, ^21^, ^22^ Unrepaired DSBs can result in cell death, while incorrectly repaired DSBs can induce gene mutations, loss of genetic information, and tumor formation.^14^^,^^23^^,^^24^ The process of DSB repair is complex, involving multiple hierarchical steps, a myriad of repair factors, and various post-translational modifications working in coordination to ensure faithful repair. Understanding the cause of DNA damage and the mechanisms of damage repair is crucial for designing targeted treatments for damage-related diseases. Researchers have discovered that R-loops can induce DNA damage events and the presence of RNA:DNA hybrids at DSB sites is significant.^25^, ^26^, ^27^ However, the formation, resolution, and function of the RNA:DNA hybrid and their contribution to genome instability remain unclear, the question of whether R-loops serve as DNA repair regulators or are merely by-products is still ambiguous.^28^, ^29^, ^30^, ^31^ The connection between R-loops and DNA damage repair will be a focal point of this review, analyzing the presence of RNA:DNA hybrids at DSB sites and evaluating their potential influence on the repair process. By synthesizing the current body of knowledge and identifying gaps in our current understanding, this review aims to provide insights into the intricate relationship between R-loops and genome instability. Ultimately, a comprehensive understanding of R-loop biology and its involvement in DNA damage repair pathways will contribute to the development of targeted strategies for the treatment of damage-related diseases.

## R-loops: from their discovery to modern research advancements

R-loops were first described in 1976. In 1994, the presence of the R-loop *in vivo* was demonstrated by analyzing plasmids isolated from *E. coli* carrying topoisomerase mutations.^32^ The development of S9.6 antibodies that specifically bind to R-loop structures in the mid-1980s opened new avenues of research.^33^

So far, various strategies have been developed to explore R-loop dynamics and functions. In cellular imaging experiments, S9.6 antibody is commonly used for detecting R-loops. However, due to S9.6's high affinity for rRNA and double-stranded RNA (dsRNA), careful interpretation of imaging results is essential.^34^ To mitigate the bias of the S9.6 antibody towards dsRNA, researchers have employed catalytically inactive human RNase H1 tagged with green fluorescent protein (GFP-dRNH1) for subsequent imaging work. This approach enhances the affinity and accuracy of measuring R-loop dynamics in live cells.^35^ Additionally, RHINO, a novel tool, was developed to improve live-cell imaging of R-loops later. RHINO's innovative design includes three copies of the RNA:DNA hybrid binding domain from human RNase H1, linked together by optimized linkers and fused to a fluorescent protein. This configuration allows for real-time live-cell imaging of R-loops, offering a more precise and dynamic method to study these complex structures.^36^

For DNA-level characterization of R-loops, researchers have utilized sodium bisulfite treatment, traditionally employed for DNA methylation pattern analysis by converting unmethylated cytosines into uracils. This method effectively maps the single-stranded DNA component of R-loops, although it faces limitations in genome-wide mapping.^37^ The discovery of endogenous R-loops, combined with rapid advancements in gene sequencing technology, has significantly propelled R-loop research forward since the early 2000s.^38^, ^39^, ^40^ Techniques such as DRIP-seq have been instrumental in this progress. DRIP-seq uses the S9.6 antibody to precipitate DNA-RNA hybrids, followed by high-throughput sequencing to analyze the structure and sequence information of R-loops.^34^^,^^41^^,^^42^ Based on this, DRIPc-seq was developed and offers accurate localization of R-loops in various cell populations with near-base-pair resolution and strand specificity.^41^^,^^43^ Another method, ssDRIP-seq, employs single-stranded DNA-linked library preparation to distinguish specific DNA strands for genome-wide R-loop identification.^44^^,^^45^ Since all these are S9.6 antibody-dependent strategies. To improve the S9.6 antibody in R-loop detection and offer an alternative research tool, R-ChIP was introduced.^48^ In R-ChIP, a mutant form of RNase H1, which can bind to RNA:DNA hybrids but cannot degrade them, is employed. The R-ChIP technique provides a way to specifically enrich and identify R-loop-containing DNA regions, which can then be analyzed further, for example, by sequencing, to determine their genomic locations and potential roles in various biological processes or diseases. Additionally, SMRF-Seq (single-molecule R-loop footprinting sequencing), BisMapR (bisulfite mapping of R-loops), MapR, R-loop cut, and tag, have been developed for R-loop mapping^46^, ^47^, ^48^, ^49^, ^50^, ^51^ (Table 1).

**Table 1:** R-loop detection strategies.

Method	Key characteristics	Antibody-dependent	Reference
DR-IP	High-through put; high-input material; not stranded; specificity for RNA:DNA hybrids is uncertain	Yes	53
DRIPc-seq	High-through put; strand specificity; high-input material	Yes	41
Bis-DRIP	High-resolution; strand specificity; less suited for genome-wide studies	Yes	54
ssDRIP-seq	High efficiency; strand specificity; high-input material	Yes	44
S1-DRIP	High-through put; high-input material; not stranded	Yes	55
R-ChIP	High-through put; stable cell line needed; inability to effectively detect R-loops in terminator regions; identify fewer R-loop forming regions than S9.6 dependent method	No	56
SMRF-seq	High resolution; strand specificity; less suited for genome-wide studies	No	47
MapR	Genome-wide; high-throughput; sensitive; native; not stranded; inability to effectively detect R-loops in terminator regions; identify fewer R-loop forming regions than S9.6 dependent method	No	51
R-loop CUT&Tag	High-resolution; less materials; no strand information	Both	50
BisMapR	High-resolution; genome-wide mapping; strand specificity; less suited for genome-wide studies	No	48

To provide detailed information on R-loops deeply and help researchers find the rules of R-loops formation. R-LoopBase, a comprehensive database was designed later. R-LoopBase extensive collection of experimentally validated R-loop data across various species offers a valuable resource for researchers studying gene regulation, genome stability, and DNA repair mechanisms. Additionally, the database includes a user-friendly interface with advanced search capabilities, visualization tools, and data integration options, facilitating easy access and analysis of R-loop information. However, despite its strengths, R-LoopBase has certain limitations. The database may not cover all known R-loops due to the dynamic and transient nature of these structures, and there might be biases toward well-studied species and cell types. Furthermore, the accuracy of the data is dependent on the quality of the underlying experimental techniques, which can vary and introduce potential errors.^52^

Although different R-loop mapping and analysis methods have their respective advantages and disadvantages, these methodologies enhance our fundamental understanding of R-loop biology and pave the way for potential diagnostic and therapeutic applications, especially in diseases where R-loop dysregulation plays a crucial role.

Today, the study of R-loops stands at an exciting crossroads. The continued development of more refined techniques promises to deepen our understanding of these complex structures. Research is now focused on deciphering the precise mechanisms of R-loop formation and resolution, their role in disease pathology, and potential therapeutic applications. As we advance, the story of R-loops continues to evolve, offering new perspectives and challenges in the field of genomic research.

## Mechanisms of R-loop formation and their implications for genome integrity

The formation of R-loops is currently believed to occur through two accepted mechanisms: i) after the nascent RNA is released from the transcription complex, it re-anneals with the template strand behind the mobile RNA polymerase, resulting in R-loop formation; ii) alternatively, the nascent RNA remains hybridized to the DNA template, extending directly within the transcription bubble to form the R-loop. The results of multiple studies do not strongly support the second mechanism.^32^^,^^33^

*In vitro* experiments have demonstrated that DNA and RNA oligonucleotides can form stable R-loops through high-temperature annealing, and two-dimensional gel electrophoresis has revealed R-loop formation during transcription-driven internal replication events in yeast.^3^^,^^57^^,^^58^ The formation of R-loops is regulated by various factors that promote the hybridization of new RNA with DNA template strands. These factors include DNA sequences that are more prone to hybridize with RNA,^59^ breaks in the non-template DNA strand,^60^ negatively supercoiled DNA structures that facilitate DNA unwinding,^61^ and non-canonical DNA structures.^62^^,^^63^ G-rich RNA sequences have a higher propensity to form stable R-loops, relying less on template supercoiling. Examples of G-rich RNA-driven R-loops include those formed during transcription of G-rich immunoglobulin class-switching regions on linear DNA templates.^37^^,^^63^^,^^64^ In terms of DNA sequences, the rG/dC sequence is more stable than dG/dC and rC/dG double-stranded sequences, and it forms the most stable RNA:DNA hybrids.^60^ When this DNA sequence is transcribed, the aforementioned RNA-DNA duplex can contribute to R-loop formation, with the tendency to form R-loops influenced by GC skew (*i.e.*, the relative abundance of guanine/cytosine in the non-template strand) due to the presence of guanine in the non-template DNA strand.

**Figure 1:**
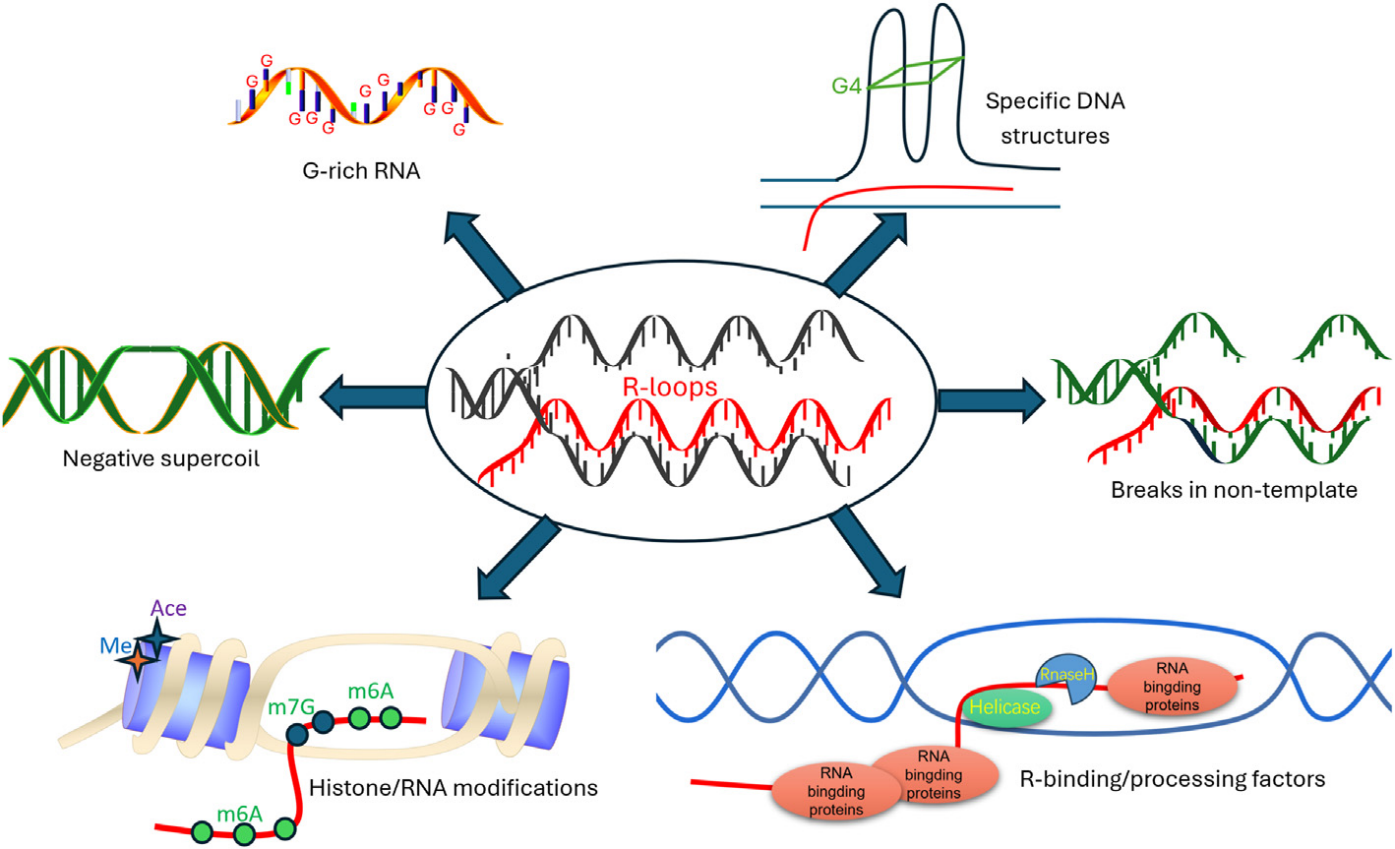
The factors contributing to R-loop formation. Several factors contribute to R-loop formation, including RNA components, specific DNA structures, histone and RNA modifications, RNA-binding proteins, and helicases.

However, the extent to which DNA sequence alone dictates R-loop formation is debated. Some studies suggest that chromatin structure and epigenetic modifications play equally or more significant roles in determining R-loop stability and formation. For instance, R-loops in the heavy chain origin regions of human and yeast mitochondria depend not only on G-rich RNA but also on negative supercoiling of the DNA template.^65^^,^^66^ Methylation of histones, such as H3K9me3 (trimethylation of lysine 9 on histone H3), is associated with a more compact chromatin state and is generally believed to suppress R-loop formation. However, recent studies have shown that certain methylation marks might also facilitate R-loop formation under specific conditions.^67^ Conversely, histone acetylation, which is associated with an open chromatin state, has been shown to promote R-loop formation. For example, acetylation of H3K27 (lysine 27 on histone H3) can lead to a relaxed chromatin structure, making the DNA more accessible for RNA:DNA hybrid formation.^68^ Given the inhibitory role of nucleosomes on R-loop formation and the dynamic nature of chromatin marks, the interactions among R-loops, nucleosomes, and chromatin modifications are complex and evolving. The relationship between R-loops and specific chromatin modifications, such as histone alterations, may therefore be subject to temporal variability, depending on when and how they form, as well as the state of the surrounding chromatin. This complexity underscores the importance of considering these modifications in research on R-loop formation and their implications for health and disease (Fig. 1).

In yeast and other organisms, ribosomal DNA (rDNA) and transposons can be prone to forming R-loops, which can lead to DNA damage due to their high transcription frequency.^69^^,^^70^ Hybrids that accumulate due to transcription in sub-telomeric regions containing telomere repeat RNA (TERRA) undergo recombination to regulate telomere maintenance.^71^

PrimPol, a DNA polymerase of the AEP superfamily, plays a vital role in tolerating DNA damage and facilitating discontinuous leading strand synthesis, even in the absence of repaired DNA. This polymerase is capable of bypassing various forms of DNA damage, such as G4 structures, chain termination nucleotide analogs, and other damaged sites, thereby restarting DNA replication. This process reduces the exposure of single-stranded DNA and limits R-loop accumulation.^72^^,^^73^ Furthermore, the formation of an R-loop may not necessarily require co-transcription of the same region. The intramolecular G-quadruplex (G4) structure, which allows continuous complementary base pairing between G-rich RNA and C-rich DNA bases, may contribute to the formation of trans-induced R-loops. Trans-induced R-loops are more likely to pose a threat to genome integrity compared with cis-formed R-loops. While cis-induced R-loops can only form within the region of RNA transcription, trans-induced R-loops can occur at multiple locations in the genome, leading to multiple unstable “hot spots”.

In addition to the previously mentioned formation and regulation of R-loops, several types of RNAs, including long non-coding RNAs (lncRNAs), circular RNAs (circRNAs), and enhancer RNAs (eRNAs), have been identified as significant contributors to the R-loop metabolism process. Up to now, increasing evidence supports the pivotal regulatory roles of lncRNAs in R-loop formation, where unchecked lncRNA transcription can perpetuate R-loop formation, thus posing a threat to genomic stability.^74^ For instance, methyl-6-adenine (m6A) modification of the lncRNA TERRA by methyltransferase 3 (METTL3) enhances R-loop formation and aids in maintaining telomere length^75^; APOLO lncRNA encourages R-loop formation at promoter regions, influencing transcription and modifying the three-dimensional structure of local chromatin across multiple distal loci^76^^,^^77^; in the same vein, lncRNA VIM-AS1 stabilizes R-loops at the VIM (vimentin) promoter region, reducing chromatin condensation.^78^ Furthermore, lncRNA HOTTIP-mediated R-loop formation plays critical roles in the regulation of genome architecture and gene expression, particularly in the context of CTCF (CCCTC-binding factor)-binding sites. HOTTIP facilitates the formation of R-loops at a subset of these sites, reinforcing CTCF boundary function and maintaining topologically associated domains, which are essential for regulating gene transcription necessary for leukemogenesis.^79^ Notably, another study has highlighted that CTCF binding sites are enriched with R-loops and G-quadruplex structures.^80^ These structures not only facilitate CTCF binding but also promote chromatin looping interactions, thereby playing a crucial role in three-dimensional genome organization and gene expression.

These discoveries prompt several important questions for further investigation. Firstly, what are the function differences between lncRNA-mediated R-loops and polymerase R-loops and G-quadruplex structures at CTCF boundary regions? Understanding this specific correlation and the implications in diseases could reveal potential mechanisms by which lncRNAs contribute to abnormal gene expression and disease progression. Secondly, do lncRNA and RNA polymerase-mediated R-loop formations, along with G-quadruplexes, interact to co-regulate gene transcription? Overall, these findings highlight the critical role of R-loops in CTCF binding and gene expression. The key issues are i) the relationship between lncRNA-mediated R-loops at CTCF boundary regions and specific diseases, and ii) the cooperative role of lncRNA and polymerase-mediated R-loops and G-quadruplexes in gene transcription regulation. Addressing these questions will enhance our understanding of the complex mechanisms of gene regulation and may offer new approaches for treating related diseases. Specifically, the discovery that lncRNA HOTTIP-mediated R-loops at the CTCF boundary functions sheds light on how disruptions in these processes might lead to diseases like leukemogenesis. Aberrant R-loop formation at CTCF sites could potentially disturb the delicate balance of gene expression, contributing to the onset and progression of various cancers and other genetic disorders. Furthermore, targeting these specific interactions between lncRNA-mediated R-loops and G-quadruplex structures could prove effective in modulating gene expression patterns that are dysregulated in diseases. For instance, small molecules or antisense oligonucleotides designed to stabilize or disrupt these structure formations might restore normal gene expression and genome stability.

In contrast, certain lncRNAs, such as TUG1 (taurine up-regulated 1), inhibit R-loop formation at specific sites by interacting with DHX9 (DExH-box helicase 9).^81^ Meanwhile, circRNAs, known for their covalently closed-loop structure, defy traditional roles as miRNA sponges by also engaging in R-loop formation. This involvement is proposed to contribute to their regulatory capacities, including transcriptional control and participation in the DNA damage response, thus unveiling a new dimension of their functionality.^82^, ^83^, ^84^, ^85^, ^86^, ^87^, ^88^, ^89^ Furthermore, eRNAs, which are transcribed from enhancer regions, contribute to gene expression activation by recruiting transcription factors and regulatory proteins to enhancers through R-loop formation, thereby promoting target gene expression and modifying the local chromatin environment to support transcriptional activation.^90^, ^91^, ^92^

Future research on non-coding RNAs and their involvement in R-loop formation is crucial for elucidating the complex molecular mechanisms that underpin their influence on genomic stability, transcription regulation, and chromatin dynamics. Emphasizing the need for a systematic characterization of lncRNA-induced R-loops, research should leverage high-throughput technologies to map these structures across various conditions and identify novel lncRNAs and their genomic targets. Additionally, the functional impact of circRNA and eRNA-mediated R-loops on gene expression and chromatin architecture requires further exploration. There is also a compelling need to explore the therapeutic potential of modulating R-loop formation in diseases characterized by genomic instability, opening avenues for RNA-based therapeutics and small molecule inhibitors. Finally, understanding the crosstalk between different types of non-coding RNAs and their collective influence on R-loop dynamics could unravel complex regulatory networks essential for cellular function and disease progression.

Overall, R-loop formation is regulated through various mechanisms and a comprehensive understanding of R-loop formation and its regulation is foundational for advancing our knowledge of cellular processes, improving disease diagnosis and treatment, and unveiling new avenues for research in genetics, molecular biology, and biomedicine.

## R-loop distribution: implications and insights

R-loops are predominantly distributed within gene bodies, occurring more frequently in gene promoters and transcription termination regions.^93^ Mammalian cell gene promoters often contain CpG islands, and the presence of R-loops on un-methylated CpG islands is associated with low DNA methylation levels. R-loops can inhibit DNA methylation in the promoter region by blocking the binding of DNA (cytosine-5) methyltransferases to DNA.^53^ This inhibition of methylation facilitates transcription by preventing methylation-mediated gene silencing, thereby promoting transcription. R-loops are also enriched in G-rich terminator elements.^2^ These R-loops function to terminate RNA polymerase II (RNAPII) extension downstream of the polyadenylation sequence. R-loops on CpG islands can trigger antisense transcription, leading to the formation of double-stranded RNA. This, in turn, recruits the RNA interference machinery and establishes repressive heterochromatin through H3K9me2 labeling to enhance RNAPII termination.^2^ During productive transcription elongation, RNA polymerase II may introduce negative supercoiling in the DNA template, and the formation of R-loops helps absorb the energy of negative supercoiling, relieving associated topological stress and restoring the DNA molecule to a lower-energy state of partial or complete relaxation.^94^

R-loops in the promoter region of genes have been found to play a role in promoting partial gene transcription. The single-stranded DNA present in the R-loop structure has the potential to facilitate antisense RNA transcription without the need for general transcription factors (GTFs) to unwind the DNA double strand. The R-loop often acts as a promoter element for producing antisense lncRNAs.^95^ In *S. cerevisiae*, it was observed that the GAL lncRNA forms an R-loop structure. The DEAD-box RNA helicase Dbp2 was found to regulate this R-loop, enhancing gene transcriptional activity.^96^ Similarly, the VIM gene, involved in regulating nuclear tissue integrity, forms an R-loop with the promoter region and transcriptional initiation site. The antisense lncRNA VIM-AS1 recruits nuclear factor-κB (NF-κB) to regulate the expression of VIM. Transcription of VIM-AS1 promotes R-loop formation, enhances the binding of the transcriptional activator NF-κB, and influences the expression of the VIM gene.^78^ Another example is the antisense lncRNA TCF21 (transcription factor 21) antisense RNA inducing promoter demethylation (TARID), which forms an R-loop with the promoter DNA of the tumor suppressor gene TCF21. The stress response protein GADD45A (growth arrest and DNA damage-inducible alpha) associates with TARID to recruit the methylcytosine dioxygenase TET1 (tet methylcytosine dioxygenase 1), inducing local DNA demethylation and activating the expression of TCF21.^97^

In *Arabidopsis*, the lncRNA auxin-regulated promoter-loop (APOLO) activates growth hormone-responsive genes. APOLO recognizes specific motifs in the promoter region of its target genes and forms R-loops in *trans*. By directly complementary base pairing with nucleic acid sequences, APOLO interacts with multiple distal target genes and anchors to their promoter regions. The single-stranded APOLO RNA acts as a decoy for the polycomb factor-like heterochromatin protein 1 (LHP1), thereby promoting the expression of target genes. The levels of APOLO influence the formation of R-loops and the transcriptional activity of these distal genes, coordinating the expression of growth hormone-responsive genes involved in *Arabidopsis* lateral root formation, such as WAG2 and AZG2.^76^

Another recent report demonstrates that R-loops form in the promoter-proximal region to regulate the termination of transcription by recruiting the SOSS-INTAC complex to these sites.^101^^,^^102^ Specifically, the SOSS component SSB1 recognizes single-stranded DNA (ssDNA) formed by R-loops through its intrinsically disordered regions. This recognition process facilitates the recruitment of the entire SOSS-INTAC complex, which in turn prevents the accumulation of R-loops that could lead to genome instability and increased chromatin accessibility.^98^^,^^99^

Crucially, SSB1's ability to form liquid-like condensates enhances the efficiency of the SOSS-INTAC complex in mitigating R-loop accumulation. This balance in R-loop levels is essential for protecting DNA from damage. The prevalence of intrinsically disordered region domains in the R-loop interactome further suggests a broader intersection between R-loops and liquid–liquid phase separation (LLPS). LLPS likely regulates R-loop formation, and conversely, R-loops may play critical roles in the formation of LLPS.^100^

These insights raise intriguing possibilities for future studies on the interactions between R-loops and LLPS, particularly at DNA damage sites where R-loops are crucial for DNA repair and many repair proteins participate in the DNA repair process through LLPS. Moreover, it remains to be determined whether the mechanism of SOSS-INTAC recruitment to R-loops is uniform across different regions of R-loop formation.

## R-loop formation and role in response to DNA double-strand breaks

The formation of R-loops can be promoted by various mechanisms in response to DNA DSBs. Transcriptional stalling induced by DSBs, as well as the presence of lncRNAs that are complementary to the ssDNA end generated during double-strand repair, and DNA end transcription by RNA polymerase II (Pol II), can all contribute to R-loop formation.^101^, ^102^, ^103^, ^104^ Recent evidence suggests that RNA Pol II is not recruited *de novo* at intergenic DSB sites,^105^ and RNA accumulation occurs primarily at DSBs located within gene sequences.^103^ Within gene bodies, DSB-induced RNA:DNA hybrids are generated through unconventional *de novo* bidirectional transcription with the DSB ends acting as promoters.^104^^,^^106^^,^^107^ This unique transcription mechanism highlights the dynamic nature of the genomic response to damage.

In mammalian cells, the RNA helicase DDX1 (DEAD-box helicase 1) has been observed to form R-loop-dependent foci following exposure to ionizing radiation. This finding indicates that DSBs can promote hybridization between RNA and the template DNA strand, contributing to the complex network of DNA damage response.^108^ Alternately, RNA Pol II can initiate transcription independently when free 3′ OH groups are present in the DNA, hence the accumulation of RNA Pol II at DSB sites is closely associated with the formation of R-loops near DSB ends, playing a crucial role in the cellular response to DNA damage.^109^

**Figure 2:**
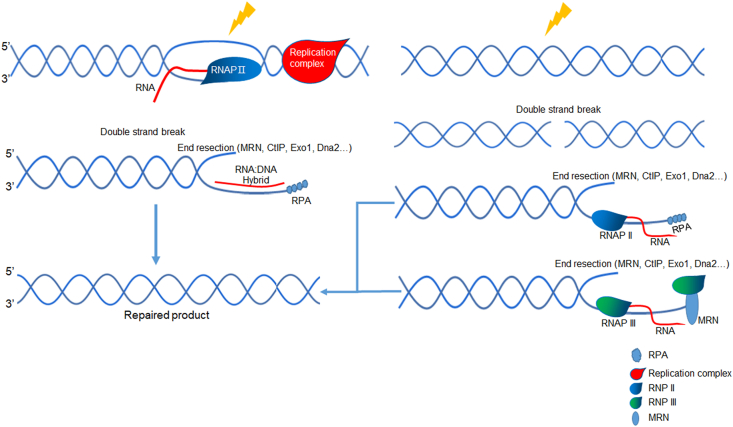
Mystery of RNA:DNA hybrid formation at double-strand break (DSB) site. When DNA damage occurs in transcriptionally active loci, pre-existing RNA molecules hybridize with the single-stranded DNA generated by DSB end resection (left), forming an RNA:DNA hybrid that protects from excessive resection. Both RNA polymerase II and RNA polymerase III are involved in the formation of RNA:DNA hybrids at the DSB site. RNA polymerase II is recruited directly or indirectly to the DSB site for transient RNA transcription, while RNA polymerase III is recruited to the DSB site in an MRN-CtIP-dependent manner. The RNA:DNA hybrid at the DSB site assists in maintaining the appropriate length of the single-stranded DNA, thus facilitating the repair process.

Furthermore, it has been reported that RNA:DNA hybrids are abundant in genes transcribed by RNA Pol III in fission yeast. RNA Pol III is responsible for forming RNA:DNA hybrids at DSB sites in a manner that depends on the MRN complex and CtIP. These newly formed RNA:DNA hybrids help protect the 3′ overhang from excessive resection, thereby promoting the efficiency of homologous recombination repair.^110^ This protective role of R-loops in homologous recombination repair highlights their importance in maintaining genomic integrity following DNA damage (Fig. 2).

Beyond its direct involvement in DNA repair processes, the interplay between R-loop modifications and their impact on genome instability remains a compelling research focus. Accumulated evidence indicates that RNA modifications such as m6A and methyl-5-cytosine (m5C) play critical roles at DNA damage sites.^111^^,^^112^ It is inevitable to explore how these RNA modifications contribute to the biological functions of R-loops and their impact on genome instability. Recent research emphasizes the significance of m6A modification in R-loops for maintaining genome stability. Specifically, METTL3-mediated m6A modification aids the efficient recruitment of RNase H1 during DNA damage repair and regulates transcription termination to preserve transcriptome integrity.^113^^,^^114^ Additionally, METTL3 has been shown to enhance homologous recombination repair by regulating RAD51 expression, a crucial factor in the homologous recombination repair pathway, or by influencing the recruitment of other homologous recombination repair factors.^112^^,^^115^ Given that m6A modifications have been found to promote R-loop formation,^116^^,^^117^ and are rapidly and transiently induced at DNA damage sites following ultraviolet exposure,^118^ questions arise about whether m6A modifications respond similarly to other forms of DNA damage, thus playing a broader role in maintaining genome integrity. Moreover, a study by Yang et al revealed that m5C modifications in RNA within R-loops favor homologous recombination over alternative non-homologous end-joining repair pathways. This preference is mediated through the regulation of m5C-induced poly(ADP-ribose) polymerase 1 (PARP1) suppression, which shifts the repair pathway choice by limiting PARP1's activity at damage sites and limiting the recruitment of downstream non-homologous end-joining repair factors.^119^

In the future study, it will be interesting to explore how these RNA modifications in R-loops affect the recruitment and activity of repair factors and the cross-talk of RNA modifications with RNA polymerase, particularly at DSB sites, where both RNA polymerase II and III contribute to R-loop formation. Additionally, exploring the influence of other RNA modifications on R-loop formation and genome instability warrants extensive investigation. These modifications include N7-methylguanosine, 2′-O-methylation, 5-hydroxymethylcytidine, N4-acetylcytidine, and inosine. Exploring these diverse RNA modifications could unveil novel regulatory mechanisms of R-loops in genome stability and provide new insights into the molecular orchestration of DNA repair pathways. Such findings could potentially lead to the development of innovative therapeutic strategies targeting RNA modifications in the context of cancer and other genetic disorders where DNA repair and genome stability are compromised.

## From formation to resolution: the multifaceted role of R-loops in genome stability and DNA repair

To maintain a balance in R-loop metabolism and prevent the detrimental consequences of persistent and unplanned R-loops, cells employ various mechanisms for R-loop resolution. A key player in this process is RNase H, a conserved enzyme adept at specifically degrading the RNA component of RNA:DNA hybrids.^120^ Additionally, the ssDNA-binding protein replication protein A (RPA) competes with R-loop formation by binding to ssDNA at the stationary replication fork and DNA damage sites. Interestingly, RPA has been reported to interact with RNase H1 and colocalize with R-loops in cells. Mutations that disrupt RPA binding to RNA lead to R-loop accumulation and genomic instability.^121^^,^^122^ A suite of RNA-dependent ATPases, including SETX, FANCM, Sen1, Mph1, AQR, DDX19, Pif1, DDX23, DDX1, Dbp2, and Sgs1, have been shown to have unwinding activity for DNA:RNA hybrids. Depletion of these ATPases results in the accumulation of DNA:RNA hybrids, emphasizing their critical role in maintaining R-loops balance.^123^, ^124^, ^125^, ^126^, ^127^, ^128^ SETX and AQR, which belong to the protein subfamily containing the conserved DEAxQ-like domain, exhibit RNA/DNA helicase activity. Deletion of the Sen1 gene in yeast, a homolog of SETX, leads to genome-wide R-loop accumulation, increased transcription-dependent recombination, and DNA damage foci.^69^^,^^129^ DDX19, an mRNA exporter, and DDX21, a DEAD-box helicase essential for rRNA gene transcription, also play vital roles in resolving R-loops, with DDX21's activity enhanced through SIRT7 mediation.^130^^,^^131^

Mutations in the BRCA1 (breast and ovarian cancer susceptibility protein 1) and BRCA2 genes, which are associated with an increased risk of breast, ovarian, pancreatic, and prostate cancers, can cause Fanconi anemia and are linked to R-loop stabilization. RPE-1 retinal pigment epithelial cells, carrying BRCA1 mutations, exhibit R-loop accumulation, which is a hallmark of cell transformation.^132^ Detailed analysis of R-loop distribution in different breast cell types has revealed that BRCA1 mutation carriers tend to accumulate more R-loops, particularly at the 5′ and 3′ ends of genes.^101^ BRCA1 is enriched in a subset of transcription termination regions where R-loops accumulate, and it mediates the recruitment of helicase SETX.^133^^,^^134^ Interestingly, R-loop accumulation was not observed in Rad51 knockdown cells, suggesting that BRCA2 may eliminate R-loops independently of DSB repair while playing roles in RAD51 loading, interstrand DNA crosslink repair, replication fork stability, and cell division.^132^

In summary, a diverse array of factors involved in DNA replication and repair plays a pivotal role in maintaining genomic stability. The intricate interplay between these factors and R-loops highlights the multifaceted nature of R-loop biology. Understanding these mechanisms is crucial for deciphering the complex dynamics of genome stability and the potential therapeutic targets within these pathways for diseases where R-loop dysregulation is a contributing factor.

## Decoding R-loops: their role in DNA damage and repair pathway choices

R-loops serve as an intermediate involved in regulating various aspects of genome dynamics. However, persistent or excessive production of R-loops can lead to transcription-replication collisions (TRCs) at the replication fork, resulting in DNA DSBs and genome instability, even compromising DNA repair.^135^, ^136^, ^137^, ^138^ R-loop formation can also contribute to DNA damage at specific DNA sequences, such as IgH S region.^126^ The formation of R-loops promotes the accumulation of Fanconi anemia factors (FANCD2, FANCA, FANCM, and BRCA2) at fragile sites,^139^ and the DEAD-box RNA helicase DDX47 is recruited to interact with FANCD2 to resolve the R-loops. As mentioned before, BRCA1 is enriched in transcription termination regions and eliminates R-loops by recruiting the helicase SETX.^134^ However, the presence of R-loops can also cause RNAPII stalling, which can further impede BRCA1 and BRCA2 function, resulting in further accumulation of R-loops and DNA damage.^140^ This suggests the presence of a sophisticated negative feedback mechanism involving BRCA factors in the regulation of R-loops. Pre-existing R-loops can also impact the DNA repair process itself. The two major DSB repair pathways, homologous recombination and non-homologous end joining are affected by the presence of R-loops, particularly in terms of altering the efficiency of end resection, a step critical for determining repair pathway choice. In fission yeast, R-loop formation prevents excessive resection at DSBs, however, R-loop removal is also necessary for the effective binding of the single-stranded DNA-binding RPA complex.^104^ Knockdown of RNase H1, an enzyme involved in R-loop metabolism, stabilizes RNA:DNA hybrids around the DSB site and severely impairs the recruitment of RPA complexes. However, overexpression of RNase H1 disrupts the stability of these hybrids, resulting in excessive DNA resection, increased RPA recruitment, and significant loss of repetitive regions surrounding the DSB site.^141^

The effects of R-loops on DNA repair depend on the context and the specific DNA repair pathway involved (Table 2). R-loops facilitate homologous recombination known as transcription-associated homologous recombination repair. In this process, RNA:DNA hybrids at DSBs recruit Rad52, which then recruits XPG proteins for R-loop processing.^142^ Beyond XPG-mediated cleavage of the R-loop structure, the absence of AQR or SETX, or inhibition of topoisomerase I can lead to the formation of R-loops, which subsequently causes DNA DSBs through the action of nucleotide excision repair endonucleases XPF and XPG. Specifically, these R-loop-induced DSBs rely on the transcription-coupled nucleotide excision repair (TC-NER) factor Cockayne syndrome group B (CSB), highlighting the involvement of the TC-NER pathway in maintaining genome stability under such conditions.^143^ ATR and ATM protein kinases, key players in the DNA damage response and maintenance of genome stability, are involved in regulating replication stress and DNA DSBs resulting from replication fork stalling, respectively.^144^ R-loop-induced replication fork arrest activates the ATR signaling pathway, while DSBs arising from replication fork collapse activate the ATM signaling pathway.^145^ Studies on TRCs have shown that R-loops have a more pronounced effect on head-on (HO) TRCs compared with co-directional TRCs.^9^ It has been demonstrated that HO TRCs lead to R-loop accumulation, disrupting transcription and causing genomic instability in human cells. HO TRCs specifically activate ATR, whereas co-directional TRCs specifically activate ATM. The mechanism underlying the selective activation of the ATR or ATM signaling pathway by R-loops in different contexts is still not clear.^146^ ATM activation may occur when R-loop accumulation leads to DSB formation or when replication proceeds through a displaced ssDNA gap. In yeast, R-loops are present at short telomeres during the S phase, and the discovery of TRCs has had a significant impact on promoting recombination to maintain telomere length and prevent senescence, with HO TRCs promoting R-loop formation and co-directional TRCs reducing R-loops.^147^ Additionally, activation of ATR and ATM facilitates the recruitment of the helicase enzyme SETX to TRCs.^148^ Activation of ATR leads to the entry of the de-capping enzyme DDX19 into the nucleus, where it unwinds the DNA:RNA hybrid strand to alleviate TRCs.^124^ Although several studies have shed light on the role of R-loops in the TRC process, the exact mechanism is still unclear. It remains to be determined whether R-loops persist and affect repair efficiency during break-end processing following TRC-induced breaks. It is well established that R-loops regulate DNA DSB repair by influencing the efficiency of DNA end excision. In yeast, the end resection factors SAE2 and its homolog CtIP facilitate the unwinding of R-loops.^149^ On the other hand, in human cells, DNA:RNA hybrid strands can enhance the excision process.^150^ A more general regulatory mechanism involves the formation of a DNA:RNA hybrid at the DSB site, known as a DR-loop, with the displacement loop (D-loop), a crucial three-stranded DNA structure in homologous recombination. This process is facilitated by the recombination factor RAD51 interacting protein 1 (RAD51AP1), which promotes RAD51-mediated recombination activity and enhances the efficiency of homologous recombination repair.^151^

**Table 2:** Influence of R-loops on DNA repair pathways and genome stability.

DNA repair pathway	Role of R-loops in the DNA repair pathway	References
Homologous recombination (HR)	Enhances the excision process; decreases the efficiency of HR repair; TA-HRR through RAD52-mediated XPG recruitment; facilitates transcription-associated homologous recombination repair (TA-HRR); regulates the efficiency of DNA end excision; promotes RAD51-mediated HR efficiency; promotes the HR repair efficiency	70, 71, 102, 103, 149
Non-homologous end joining (NHEJ)	Decreases the efficiency of NHEJ repair pathways	149
Transcription-coupled nucleotide excision repair (TC-NER)	Associated with transcription-coupled nucleotide excision repair (TC-NER)	152
Single-strand annealing (SSA)	R-loops promote SSA efficiency by facilitating the alignment of homologous sequences during recombination	106
Break-induced replication (BIR)	Plays a role in BIR by promoting replication fork stalling and subsequent repair events	138

Overall, the role of R-loops in DNA damage and repair pathway choices is complex and multifaceted, encompassing a range of mechanisms from contributing to transcription-replication collisions and influencing DNA DSB repair, to interacting with various DNA repair pathways and factors. This intricate interplay underscores the critical role of R-loops in genome stability, where their regulation and resolution are pivotal in maintaining the delicate balance between necessary cellular processes and the prevention of genomic instability.

## R-loops in genomic instability: implications in cancer, autoimmune diseases, and neurodegeneration

For the time being, R-loops can be categorized into physiological and pathological types. Physiological R-loops are generated through specific programmed processes and are often found in specific regions. Physiological R-loops play important roles in various biological processes, including gene expression regulation,^153^ DNA repair,^104^ immunoglobulin class switch recombination,^37^ CRISPR-Cas9-induced dsDNA recognition,^154^ regulation of DNA replication processes in mitochondrial DNA, bacterial plasmids and phages,^3^ RNAi-directed heterochromatin assembly in fission yeast,^155^ and telomere formation and maintenance.^4^ In contrast, the “pathological” R-loop is characterized by its aberrant formation and the detrimental effects it has on genomic stability and cellular function. Many ways can cause pathological R-loop formation, such as dysregulation of RNA processing, high transcription activity, DNA damage and repair defects, epigenetic changes, *etc.* Several key features can be used to distinguish pathological R-loops from normal R-loops. i) Pathological R loops form excessively or in inappropriate genomic locations. ii) Pathological R-loops contribute to genomic instability. iii) Pathological R-loops can obstruct the progress of RNA polymerase during transcription and impede DNA replication. This interference can lead to TRCs, which further contribute to genomic instability. iv) They can cause dysregulation of gene expression. v) The presence of pathological R-loops can induce cellular stress responses and lead to various forms of cellular dysfunction. vi) Pathological R-loops have been implicated in a variety of diseases, including neurodegenerative disorders, cancer, and autoimmune diseases. As summarized before, the dysregulation or excessive formation of R-loops is associated with various pathological conditions: promoting transcription-replication fork conflicts, involvement in DNA excision,^156^ induction of DNA breaks, and leading to abnormal repair processes.^4^^,^^157^^,^^158^ Though pathological R-loops have been reported to correlate with many types of disease, the exact mechanisms by which they contribute to disease development and progression are still an active area of research.^157^^,^^159^^,^^160^ In the context of cancer, excessive R-loop formation leads to genomic instability, particularly in cancers with defective DNA repair mechanisms. This is clearly observed in BRCA1-deficient breast and ovarian cancers, where the loss of BRCA1 function results in increased R-loop formation, DNA damage, and chromosomal rearrangements,^140^^,^^161^^,^^162^ accelerating tumorigenesis. However, questions remain about how BRCA1 regulates R-loops genome-wide, the specific regions where BRCA1 processes R-loops, and the correlation between BRCA1's R-loop regulation function and tumor formation.^163^ Additionally, in certain conditions, the genome instability induced by R-loop accumulation can prevent tumorigenesis; for example, ablation of WDR61 can suppress cancer progression due to the inhibition of cell proliferation.^164^

So far, genomic instability, a critical factor in various human diseases, is increasingly linked to the accumulation of R-loops. In the realm of cancer, this instability is highlighted by the aberrant hypermethylation of tumor suppressor gene promoters, leading to silencing and tumorigenesis.^165^^,^^166^ Intriguingly, R-loops at gene promoters can counteract this by preventing methylation by DNA methyltransferase DNMT3B, thus sustaining gene activity.^53^ Understanding this interplay between R-loop levels and hypermethylation is vital for advancing cancer detection and treatment strategies.

In Ewing sarcoma, the expression of the EWS-FLI1 fusion protein enhances transcription, leading to R-loop accumulation. This accumulation, along with the interaction between EWS-FLI1 and BRCA1, hinders homologous recombination and contributes to genomic instability in cancer cells.^140^ Furthermore, histone H3S10 phosphorylation (H3S10P), which is associated with chromatin condensation, has also been found to correlate with R-loops.^167^ The accumulation of H3S10P is observed in yeast, *Caenorhabditis elegans*, and human cells lacking mRNA processing factors. Highly condensed chromatin regions may trigger gene silencing, replication, or transcription arrest, and ultimately contribute to genomic instability and cancer development. Beyond cancer, R-loops are also implicated in autoimmune diseases. Aicardi-Goutières syndrome (AGS), an inflammatory disorder of the nervous system, is caused by mutations in the subunit of RNase H2.^168^^,^^169^ AGS is characterized by the accumulation of ribonucleotides in DNA, resembling congenital viral infections and resulting in neurological damage.^170^ RNase H2 is composed of three catalytic subunits (2A, 2B, and 2C), and mutations in any of these subunits can cause AGS. Mutations in other DNA-related enzymes, such as ssDNA 3′–5′ exonuclease TREX1 (three prime repair exonuclease 1; DNASEIII), dsRNA-editing enzyme ADAR1 (adenosine deaminase acting on RNA 1), and dNTP triphosphohydrolase SAMHD1 (SAM and HD domain containing deoxynucleoside triphosphate triphosphohydrolase 1), have also been associated with AGS.^171^^,^^172^ Studies in yeast have shown that mutations in RNase H2 associated with AGS result in reduced RNA/DNA cleavage activity.^173^ While the involvement of R-loops in AGS pathology is suggested, further research is needed to determine the specific contributions. Neurodegenerative diseases also exhibit associations with R-loop dysregulation. Repeat expansions, central to disorders such as ataxias, amyotrophic lateral sclerosis (ALS), and nucleotide expansion disorders, often involve R-loop formation. For instance, R-loops have been detected in cells from patients with Friedreich's ataxia and Fragile X syndrome, diseases caused by expanded GAA and CGG repeats.^53^ Mutations in the RNA:DNA helicase SETX are associated with two different neurological diseases, ataxia with oculomotor apraxia type 2 (AOA2) and a juvenile form of ALS known as ALS4, highlight the role of R-loop resolution in neuronal diseases.^174^^,^^175^ SETX has been shown to regulate neuronal differentiation through fibroblast growth factor 8 (FGF8) signaling.^176^ However, the precise role of SETX in the interplay between R-loops, genome maintenance, and neuronal differentiation remains unclear.

Overall, there are multiple ways that R-loops can disrupt genomic processes, leading to compromised genome integrity which eventually causes disease. Firstly, R-loops can induce DNA breaks, particularly DSBs, which are one of the most deleterious types of DNA damage. Such breaks can lead to chromosomal rearrangements and mutations, contributing to the onset and progression of cancers and other genetic disorders. Additionally, R-loops can cause transcription-replication conflicts, where the machinery for DNA replication and RNA transcription collide on the DNA template. These conflicts not only stall replication and transcription but can also result in mutations and chromosomal instability, further aggravating disease conditions. Furthermore, R-loops can alter gene expression by influencing the transcriptional machinery either through direct physical obstruction or by modulating the chromatin state. This dysregulation of gene expression can contribute to diseases by disrupting the normal patterns of protein production, which is critical for cell function and integrity. Thus, the accumulation or unresolved R-loops can be a significant factor in the pathogenesis of various diseases, especially those related to genomic instability, such as cancer, neurodegenerative diseases, and certain inherited disorders (Table 3).

**Table 3:** R-loops and their association with human disorders.

Disease	Proposed mechanism	Impact	References
Cancer	R-loops cause genomic instability, leading to DNA damage, mutations, and chromosomal rearrangements	Drives oncogenesis and cancer progression	102, 134, 164, 181, 182, 183
Neurodegenerative diseases (*e.g.*, amyotrophic lateral sclerosis, frontotemporal dementia)	Mutations in genes like C9orf72 lead to R-loop formation, causing DNA damage and interfering with RNA processing	Contributes to neuronal death and progression of neurodegenerative diseases	157, 184, 185, 186
Friedreich's ataxia	GAA trinucleotide repeat expansions promote R-loop formation, inhibiting FXN gene transcription and reducing frataxin protein	Leads to impaired mitochondrial function and neurodegenerative symptoms	62, 187
Systemic lupus erythematosus (SLE)	Accumulation of R-loops potentially triggers an autoimmune response by exposing hidden DNA segments to immune cells.	Causes inflammation and tissue damage in SLE patients	188, 189
Aicardi-Goutières syndrome (AGS)	Mutations in genes like TREX1 lead to R-loop accumulation, activating innate immune response and causing inflammation	Causes brain and skin inflammation, characteristic of AGS	190, 191, 192
Huntington's disease	CAG repeat expansions lead to R-loop formation, disrupting gene expression and protein synthesis	Results in neurological deterioration and movement disorders	193, 194
Myotonic dystrophy	CTG repeat expansion in the DMPK gene causes R-loop formation, affecting RNA processing and protein function	Leads to muscle weakening and other systemic symptoms	195, 196
Ataxia telangiectasia	Defective DNA repair mechanisms lead to R-loop accumulation, contributing to neurodegeneration and immune system dysfunction	Causes progressive loss of movement control and immune system abnormalities	4, 27, 180

**Figure 3:**
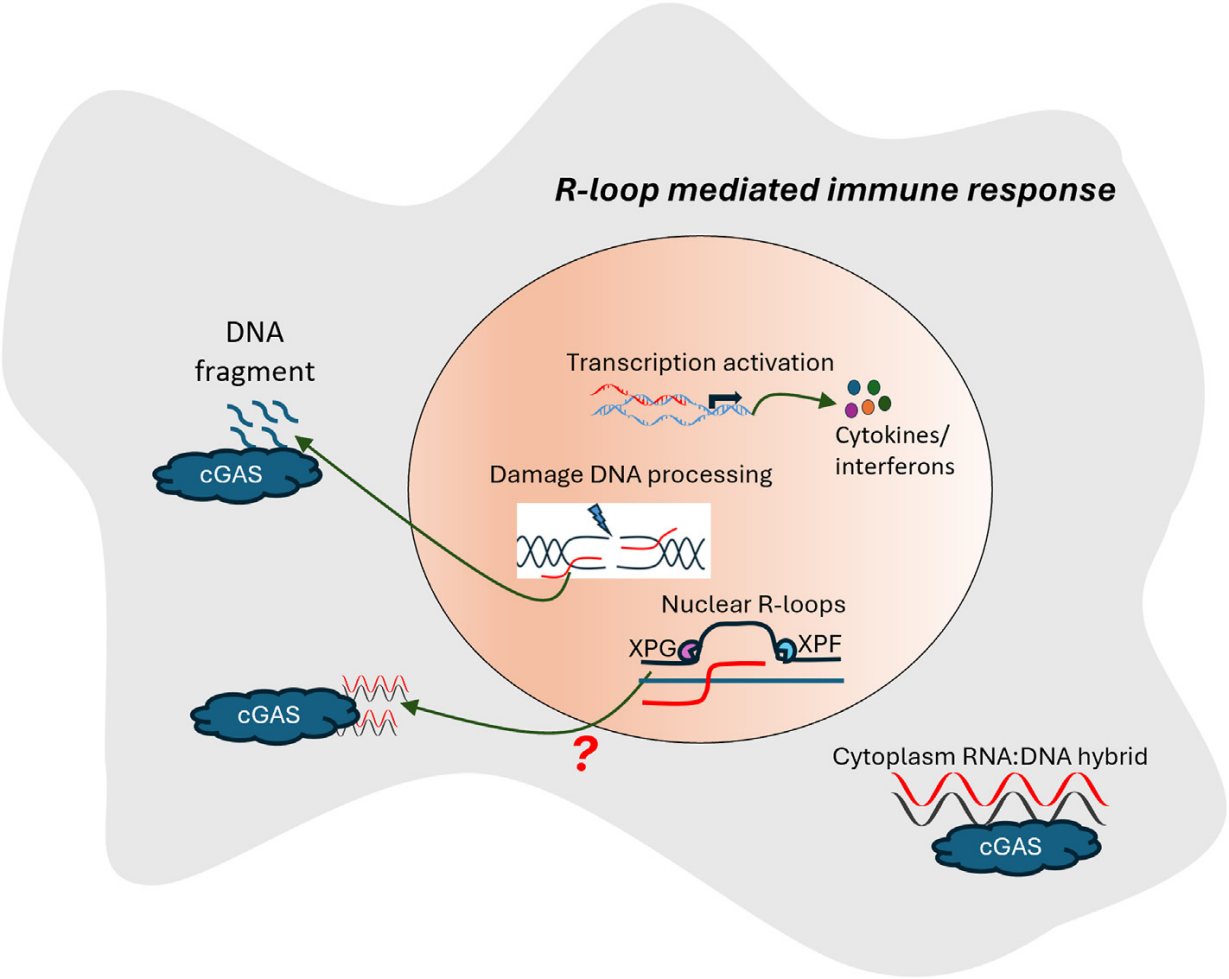
R-loop mediated immune response. Several mechanisms exist to regulate R-loop-mediated immune responses, including the recognition of cytoplasmic R-loops by cGAS (cyclic-GMP-AMP synthase), the influence of R-loops on gene expression related to immune responses, and the release of DNA or R-loops into the cytosol. The DNA damage repair can also be recognized by cGAS.

Besides, R-loops are increasingly recognized as key players in the regulation of immune signaling pathways.^177^^,^^178^ They are involved in the activation of immune responses through the modulation of gene expression in immune cells. For instance, R-loops can influence the transcription of genes critical for the innate immune response, thereby impacting the production of cytokines and interferons, which are vital for defending against infections and initiating inflammatory responses.^179^ Moreover, the accumulation of R-loops can be sensed as a form of genomic stress or damage, triggering cellular pathways that lead to the activation of immune responses. This sensing mechanism is particularly relevant in conditions like viral infections, where the presence of viral RNA can lead to the formation of aberrant R-loops, subsequently alerting the innate immune system. In cases where R-loops induce a DNA damage response, improperly processed DNA during this process may release DNA into the cytoplasm during the cell cycle. This released DNA is then detected by the DNA sensor cGAS (cyclic-GMP-AMP synthase), triggering subsequent immune response pathways. Additionally, the unresolved R-loops can contribute to chronic inflammation, a key feature in autoimmune diseases, by continuously activating immune pathways.^177^ Recently, researchers found that R-loops released from the nucleus into the cytosol can be detected by the DNA sensor cGAS, and trigger the cGAS-STING mediated immune response, though how the R-loops accumulated in the cytosol and the erasing mechanisms are still unclear^180^ (Fig. 3). Thus, the link between R-loops and innate immunity is an expanding area of research, offering new insights into how cellular nucleic acid metabolism can influence immune surveillance and the pathogenesis of immune-related disorders.

In summary, R-loop-dependent genomic instability manifests in various aspects, from cancers, neurological disorders, to immune responses. These structures, while crucial for normal cellular functions, can contribute to disease pathogenesis when dysregulated. Understanding the nature of R-loop biology in these contexts is essential for developing targeted therapies and improving patient outcomes.

## Conclusion

Our understanding of R-loop formation, resolution, and biological functions has advanced significantly in recent years. While R-loops were initially considered transcriptional by-products, we now recognize their physiological roles in various processes, including DNA replication, gene regulation, and class switch recombination.^6^^,^^95^^,^^136^^,^^197^^,^^198^ However, unraveling the complex nature of R-loops and elucidating their dual roles remains challenging.

Recent findings highlight the importance of R-loops in DNA damage response and repair, revealing both protective and harmful effects depending on the context. Numerous DNA repair factors, including BRCA1, BRCA2, CtIP, RAD52, SETX, and AQR, have been implicated in R-loop metabolism.^123^^,^^132^^,^^134^^,^^142^^,^^149^^,^^199^ Yet, the precise connection between their DNA repair functions and their capabilities in processing R-loops, particularly in the context of cancer-associated mutations in BRCA1/BRCA2, remains elusive. For example, SETX, a DNA repair factor associated with oculomotor apraxia type 2 (AOA2) and juvenile amyotrophic lateral sclerosis (ALS4), has been widely accepted as an R-loop processing helicase. Recent research， however, suggests that SETX depletion actually decreases the overall genome-wide R-loop levels, challenging the previous notion.^200^ These findings indicate that R-loop formation, resolution, and function may be dependent on spatial–temporal regulation.

The presence of R-loops at DNA DSB sites is well-documented, yet their precise role at these sites is still debated. RNA Pol II and Pol III have been implicated in DNA:RNA hybrid formation at DSB sites,^104^^,^^110^ while conflicting reports suggest transcription and nascent RNA synthesis inhibition under damage treatment.^31^^,^^70^^,^^201^, ^202^, ^203^ This discrepancy raises questions about whether RNA:DNA hybrids at DSB sites are formed from pre-existing RNA or RNA synthesized post-damage. Furthermore, understanding R-loop dynamics in transcriptionally inactive loci during DNA damage is essential for elucidating their broader functional implications.

Moreover, exploring RNA modifications in R-loops and their consequential effects on diverse cellular mechanisms, especially in DNA repair, represents a captivating and burgeoning field of study. RNA modifications such as m6A and m5C play pivotal roles in DNA damage response and genome stability. Targeting these modifications that influence R-loop formation presents a promising therapeutic strategy. For instance, m6A modification by METTL3 enhances R-loop formation and facilitates the recruitment of DNA repair proteins. Drugs that modulate RNA modifications or their interacting proteins could potentially stabilize beneficial R-loops or resolve harmful ones, offering a novel approach to disease treatment.

Despite significant research progress, numerous questions about R-loops remain unanswered, presenting potential avenues for future research. This includes the connection between R-loops and diseases. As mentioned before, mutations in the RNase H2 family have been associated with AGS, while most reports have focused on the role of RNase H2 in removing ribonucleotides from DNA, the potential role of RNaseH2 in R-loop processing and its implications for AGS require further investigation. Similarly, the extent to which SETX's R-loop resolving activity contributes to neurological diseases remains to be clarified. Additionally, the interplay between R-loops and LLPS in DNA damage repair and genome stability warrants extensive exploration. Understanding the mechanistic link between R-loops and LLPS could provide novel insights into DNA damage repair regulation and genome stability maintenance. Targeting the molecular components involved in R-loop formation and LLPS could pave the way for innovative therapeutic strategies aimed at enhancing DNA repair mechanisms. Such approaches may be particularly beneficial in treating cancers that exhibit deficiencies in DNA repair pathways. Additionally, modulating LLPS dynamics might offer new avenues for treating neurodegenerative diseases where protein aggregation plays a pivotal role. Overall, insights into the R-loop and LLPS relationship could lead to the development of novel diagnostics and therapeutics, ultimately improving patient outcomes in various genetic and degenerative disorders. Thus, mechanistic insights into R-loop formation at DSB sites, the role of pre-existing versus newly synthesized RNA in hybrid formation, and the impact of RNA modifications on R-loop dynamics are crucial for understanding the intricate regulation of genomic stability.

The translational implications of R-loop research are profound, influencing our understanding of disease mechanisms, particularly in cancers with defective DNA repair mechanisms like BRCA1-deficient breast and ovarian cancers. Excessive R-loop formation accelerates tumorigenesis through genomic instability and chromosomal rearrangements. Conversely, targeting pathways that resolve transcription-replication conflicts or using antisense oligonucleotides to modulate R-loop formation could hold therapeutic promise.

In summary, the function of R-loops in genome stability is still an evolving field of study. The roles of R-loops in disease, both direct and indirect, are still being explored. Investigating the R-loop processing functions of specific proteins, such as AQR, RNase H2, and SETX, will provide valuable insights into their contributions to disease pathology. This ongoing research is key to unlocking the full potential of R-loops in therapeutic applications and our understanding of their roles in health and disease.

## Funding

This work was supported by research funding from the 10.13039/501100001809National Natural Science Foundation of China (No. 32201061) and the 10.13039/501100007129Natural Science Foundation of Shandong, China (No. ZR2021QC083).

## CRediT authorship contribution statement

**Min Zhu:** Conceptualization, Funding acquisition, Writing – review & editing. **Xinyu Wang:** Writing – original draft. **Hongchang Zhao:** Conceptualization, Supervision, Writing – review & editing. **Zhenjie Wang:** Conceptualization, Supervision, Writing – review & editing.

## Conflict of interests

The authors declare that the research was conducted in the absence of any commercial or financial relationships that could be construed as a potential conflict of interest.
